# Association of Major Depressive Episode with Negative Outcomes of Tuberculosis Treatment

**DOI:** 10.1371/journal.pone.0069514

**Published:** 2013-07-29

**Authors:** Cesar Ugarte-Gil, Paulo Ruiz, Carlos Zamudio, Luz Canaza, Larissa Otero, Hever Kruger, Carlos Seas

**Affiliations:** 1 Instituto de Medicina Tropical Alexander Von Humboldt, Universidad Peruana Cayetano Heredia, Lima, Peru; 2 Mental Health Working Group - Universidad Peruana Cayetano Heredia, Lima, Peru; 3 Departamento de Enfermedaded Infecciosas, Tropicales y Dermatológicas, Hospital Nacional Cayetano Heredia, Lima, Peru; McGill University, Canada

## Abstract

**Background:**

Pulmonary tuberculosis (TB) persists an important contributor to the burden of diseases in developing countries. TB control success is based on the patient’s compliance to the treatment. Depressive disorders have been negatively associated with compliance of therapeutic schemes for chronic diseases. This study aimed to estimate the significance and magnitude of major depressive episode as a hazard factor for negative outcomes (NO), including abandon or death in patients receiving TB treatment.

**Methodology/Principal Findings:**

A longitudinal study was conducted to evaluate the association of major depressive episode (MDE), as measured by a 5-item version of the Center for Epidemiological Studies Depression Scale (CES-D) with NO to TB treatment. Patients with confirmed TB were enrolled before the start of TB treatment. Baseline measurements included socio-demographic variables as well as the CES-D, which was also applied every month until the end of the treatment. Death and treatment default were assessed monthly. Survivor function (SF) for NO according to MDE status (CES-D≥6) at baseline (MDEb) was estimated. Cox’s Regression was performed for bivariate analyses as well as for the multivariate model. A total of 325 patients accepted to participate in the study, of which 34 where excluded for diagnosis of MDR-TB. NO was observed in 24 patients (8.2%); 109 (37%) presented MDEb. Statistically significant difference was found on the SF of patients with and without MDEb (0.85 vs. 0.96, p-value = 0.002). The hazard ratio for NO, controlled for age, sex, marital status and instruction level was 3.54 (95%CI 1.43–8.75; p-value = 0.006).

**Conclusion:**

The presence of MDE at baseline is associated to NO of TB treatment. Targeting detection and treatment of MDE may improve TB treatment outcomes.

## Introduction

Tuberculosis (TB) still remains as one of the biggest global health problems, with 8.7 million incidence cases in 2011 [1]. Peru has one of the highest rates in Latin America, with 101 new cases per 100,000 persons and 2100 estimated cases of multidrug resistant-TB (MDR-TB) in 2011 [1], and despite the strengthening of control strategies in the last years, there are some areas in Peru with high TB burden [2] and with high prevalence of primary MDR-TB [3].

One of the biggest challenges in TB control is the adherence to the treatment under DOTS regimen (Directly Observed Therapy Short course): the treatment is daily in the first two months and then twice or three times a week during 4 months, creating many problems for the follow up. Non-adherence is related with increasing cases of MDR-TB and negative outcomes as death or clinical complications [4]. Several risk factors were associated in different settings with abandon TB treatment as poverty, drug abuse, complexity of the treatment, access to health services and mental health conditions as Depression [2], [5].

Evidence suggests that depressive symptoms are associated with lower adherence for treatment of chronic conditions, similar in therapeutic regimen to TB, such HIV [6], [7]. In 2002 a review by Wing et al suggested that even if depressive symptoms were related to poorer adherence to treatment, this do not necessarily relates to a poorer outcome, suggesting that depressive symptoms might influence both adherence and outcomes. This review found that depressed patients were less likely to be adherent to treatment due to feelings of hopelessness about therapeutic regimen, lack of social support, energy or memory [8].

The relationship between depressive disorders and TB is complex. Personal, socio-cultural and environmental factors might make persons with a TB diagnosis more prone to depressive symptoms. Factors related to depressive symptoms in TB patients in a sample of homeless in Los Angeles, USA, included physical health limitation, multiple sex partners, daily drug use, alcohol dependence and education degree below high school, while social support was found to be protective [9].

One of the problems to evaluate patient’s mental health (in this specific case, a Major Depressive Event) is the time-demanding tests. In Peru, the burden of mental health in the population is not well known [10], and the relation of the depression and TB is not well studied. The Center for Epidemiologic Studies Depression Scale (CES-D), 5-items, self-report test, performed well when validated in a Peruvian population [11]. We chose this tool to contribute with evidence on the frequency of Major Depressive Disorder (MDE) among TB patients and its association to a negative outcome (NO) in a population with high rates of TB in Lima, Peru.

## Methods

### Ethics Statement

Prior to their participation in the study, all persons went through the informed consent process, and those that accepted to participate signed the informed consent document. The research protocol was reviewed and approved by the Universidad Peruana Cayetano Heredia institutional review board and by the Local Health Department (Dirección de Salud Lima Este).

Patients who had a CES-D score compatible with suspicion of MDE were referred to the treating physician of the Peruvian National TB Program. As per national guidelines, the treating physicians are responsible to diagnose and to provide adequate management of TB co-morbidities, including depression, HIV infection, diabetes and any other co-morbidities [12].

### Study Design

We conducted a longitudinal study, the main response variable was the presence of a NO to TB treatment, which consisted on either death or discontinuation (abandon) of TB treatment without medical indication. The variable of interest, which is hypothesized to modify the hazard of presenting a negative outcome for TB therapy were symptoms suggesting MDE before the start of TB therapy, defined by a score of 6 or higher on the 5 items CES-D scale. Assessment of TB treatment outcome was made in monthly basis visit, in the routine clinical TB evaluation.

### Study Setting and Population

The study was conducted in a large district in North Lima (Lima, Peru) during October 2010 and August 2011. Participants were newly diagnosed with TB by smear and/or culture positive result for *Mycobacterium tuberculosis*, and were about to start TB treatment at their DOTS clinic. Other inclusion criteria were age ≥18 years old, no previous diagnosis or treatment for TB and non-MDR-TB. Exclusion criteria included illiteracy or any physical or mental condition that diminished the patients capability of a proper understanding during the process of the informed consent and pregnancy.

To record information during the baseline interview, a structured questionnaire containing general socio-demographic and health questions was applied by a health care worker trained for the purpose. A locally validated, 5 items version of the Center for Epidemiological Studies Depression Scale (CES-D) scale [11], [13] was completed by each participant that accepted to participate in the study. The 5 items CES-D version completed by the participants reported to have a sensitivity of 95.7%, specificity of 93.4% and correctly classify 94.7% of individuals, using a cut-off score of 6 or more. The scale also showed a good internal consistency (Cronbach’s Alpha  = 90.5; a measure where 100 is perfect consistency) [11]. This scale was completed at the beginning of the treatment (V0) and in each monthly visit until the end of the treatment (V1–V6).

### Sample Size

For the sample size calculation, we considered that those with MDE at the first visit for anti-TB treatment would have a hazard two times higher than patients without evidence of MDE during the first visit. This was a more conservative approximation than literature suggested for mental disorders such as substance abuse [14]. We also assumed a frequency of at least 20% of NO across the duration of the anti-TB treatment. With 5% probability of type I error and 20% probability of type II error, the resulting sample size was 327 persons enrolled in the study.

### Statistical Analysis

The data analysis consisted of exploratory description of interest variables using central tendency and dispersion measurements as well as the distribution of the event of interest according to the covariates. To test our main hypotheses we followed a time-to event approximation using Cox’s regression. Also, covariates of interest including age at baseline, sex, marital status and instruction level, were tested as independent variables on the Cox’s regression. Finally a multi-variate model including variables that resulted significantly (p<0.05) predictors of NO in the bivariate analysis were included along with MDE on the baseline (MDEb) measurement in a multi-variate model also using Cox’s regression.

All analyses were done using the statistical software Stata v.11.1 (Statacorp Texas, US) and the statistical power calculation was done using the PASS 2012 software (NCSS Utah, US).

## Results

### General Characteristics of the Sample

A total of 325 persons were included in the study, among these 34 participants received laboratory diagnosis of MDR-TB and were excluded for the analysis. The median age was 28 years old (IQR = 16) among participants with MDE at baseline (MDEb) and 24 (IQR = 9) among participants without MDEb. As presented in Table 1, participants varied significantly across MDEb participants and non-MDEb participants in terms of gender (p = 0.045), marital status (P-value = 0.016), education level (P-value<0.001) and employment status (P-value<0.001). As a result, persons with MDEb were older and more likely female, single, lower education level and unemployed.

**Table 1:** Sample description at baseline by main predictor variable.

Variable		MDEb*		No MDEb		
		n = 109		n = 182		
		n	%	n	%	P-value
Female		57	53%	74	41%	0.045
Marital Status	Single	50	47%	116	64%	0.016
	Cohabitant or Married	45	41%	55	30%	
	Divorced/Widowed	13	12%	11	6%	
Education Level	Incomplete Elementary	15	8%	7	4%	<0.001†
	Complete Elementary	42	29%	42	23%	
	Complete High School	37	41%	84	46%	
	Superior	14	22%	49	27%	
Alcohol Intake	Never	41	39%	76	41%	0.696
	Social events only	42	40%	63	37%	
	At least onceper month	23	21%	43	22%	
Illegal Drugs	Cannabis	9	8%	16	9%	0.893†
	Cocaine Basic Paste	12	11%	10	8%	0.107†
Employment	Unemployed	79	73%	97	53%	0.001†
	Employee	6	6%	31	17%	
	Independent Work	23	21%	54	30%	

† Fisher’s exact test.

* MDE = Major Depressive Disorder at enrollment.

### Survival Description of Negative Outcomes

**Figure 1:**
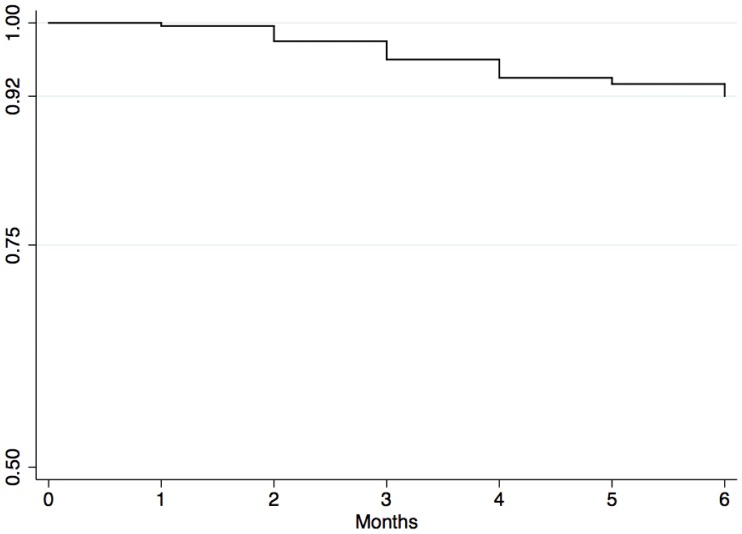
Survival Kaplan-Meier curve for Negative Outcomes of TB treatment.

Across the study, 24 participants (8.2%) abandoned the PTB treatment or died. There was a statistical difference of presenting NO across the duration of the study between participants with MDEb compared with non-MDEb (P-value = 0.003). Time series table containing detailed description of time to event for negative outcomes can be found in Table 2 and are visually presented in Figure 1.

**Table 2:** Survivor function (SF) for Negative Outcomes (NO) on TB treatment.

Time	n	Negative Outcome	Survivor Function	CI 95%
Baseline	291	–	–	–
1st MFU	291	1	0.997	0.98–0.99
2nd MFU	290	5	0.979	0.95–0.99
3rd MFU	285	6	0.956	0.93–0.98
4th MFU	279	6	0.938	0.90–0.96
5th MFU	273	2	0.931	0.89–0.95
6th MFU	271	4	0.917	0.88–0.94

CI95%: 95% Confidence interval; MFU: Monthly follow up.

### Depression Score across Time

Average depression scores and proportion of patients with a CES-D score suggesting MDEb tended to be lower the longer the patients stayed in the TB treatment, specially after the second follow up visit (Table 3). Both CES-D scores and MDEb proportion were significantly different (p<0.001) between V0 and V1 to V6.

**Table 3:** Depression measurements (CES-D mean) across visits.

	MDE at Baseline				Non-MDE at Baseline			
Time	CES-D mean	CES-D SD	Proportion	CI 95%	CES-D mean	CES-D SD	Proportion	CI 95%
Baseline	9.30	2.92	100.0%	–	2.22	1.62	0.0%	–
1st MFU	4.76	3.68	38.3%	28.9–47.7%	2.81	2.71	18.2%	12.4–23.9%
2nd MFU	3.94	3.76	31.7%	22.4–40.9%	2.22	2.82	12.8%	7.7–17.8%
3rd MFU	2.90	2.98	20.0%	11.8–28.2%	2.32	2.7	13.8%	8.5–19.1%
4th MFU	2.78	3.01	20.8%	12.4–29.4%	1.85	2.03	8.0%	3.8–12.2%
5th MFU	2.12	2.56	11.8%	4.8–18.7%	1.75	2.10	10.1%	5.3–14.8%
6th MFU	2.65	2.77	17.9%	9.5–26.2%	1.71	2.27	11.3%	6.3–16.3%

MDE: Major depressive episode; CES-D: Center for Epidemiological Studies Depression Scale - 5 items version; CI95%: 95% Confidence interval; MFU: Monthly follow up.

### Negative Outcomes and Depression

**Figure 2:**
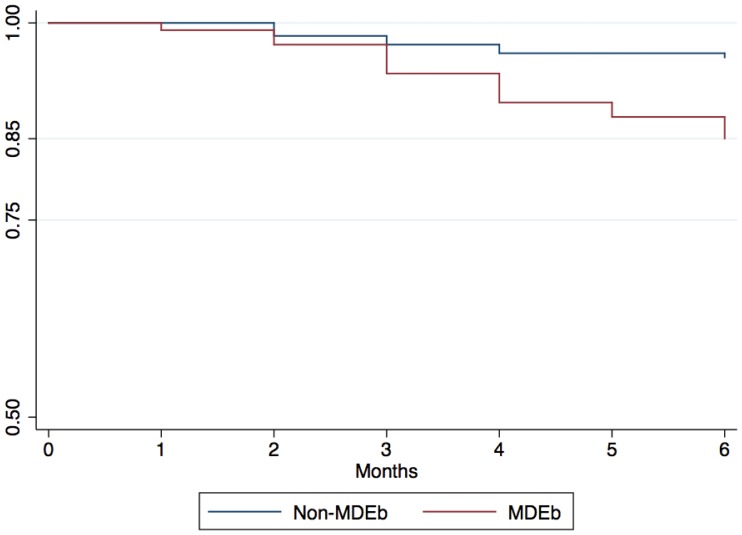
Negative Outcomes Survival Kaplan-Meier curve by MDE at baseline.

At V6, the survivor function for participants that presented CES-D scores compatible with MDEb was 85% (95%CI: 77–91%), while for persons without evidence of MDEb was 96% (95%CI: 91–98%) (Table 4 and Figure 2).

**Table 4:** Survivor Function (SF) and MDE at baseline (MDEb).

	MDE at Baseline				Non-MDE at Baseline			
Time	n at start ofinterval	NO	SF	CI 95%	n at start of interval	NO	SF	CI 95%
Baseline	109	–	–	–	182	–	–	–
1st MFU	109	1	0.991	0.94–1.00	182	0	100	–
2nd MFU	108	2	0.972	0.92–0.99	182	3	0.984	0.95–0.99
3rd MFU	106	4	0.936	0.87–0.97	179	2	0.973	0.93–0.99
4th MFU	102	4	0.899	0.82–0.94	177	2	0.961	0.92–0.98
5th MFU	98	2	0.881	0.80–0.93	177	0	0.961	0.92–0.98
6th MFU	96	3	0.853	0.77–0.91	175	1	0.956	0.91–0.98

MDE: Major depressive episode; CES-D: Center for Epidemiological Studies Depression Scale - 5 items version; CI95%: 95% Confidence interval; MFU: Monthly follow up; NO: Negative Outcome.

When using Cox’s regression to evaluate MDEb visit as predictor of negative outcomes for PBT, we found that patients with a CES-D score at baseline suggestive of MDEb had 3.46 times the hazard of presenting TB treatment abandon or death than those with no MDEb (95%CI: 1.48–8.08)(Table 5).

**Table 5:** Univariate model of MDE as hazard factor for Negative Outcome.

Variable		HR	P-value	CI 95%
MDE at baseline		3.46	0.004	1.48–8.08
Age at baseline		1.00	0.652	0.97–1.04
Female		0.79	0.579	0.34–1.82
Marital Status	Single	Reference Category		
	Cohabitant/Married	1.39	0.463	0.58–3.35
	Divorced/Widowed	1.95	0.305	0.54–6.99
Instructionlevel	Incomplete Elementary	Reference Category		
	Complete Elementary	0.78	0.767	0.16–3.89
	Complete High School	0.91	0.901	0.19–4.14
	Superior	0.87	0.865	0.17–4.47
Alcohol Intake	Never	Reference Category		
	Social events only	0.39	0.109	0.12–1.23
	At least onceper month	1.13	0.586	0.52–3.20
Illegal Drugs	Cannabis	1.55	0.477	0.46–5.20
	Cocaine Basic Paste	3.4	0.015	1.27–9.10
Employment	Unemployed	Reference Category		
	Employee	0.59	0.485	0.14–2.58
	Independent Work	0.7	0.485	0.26–1.91

MDE: Major Depressive Episode; HR: Hazard Ratio; CI 95%: 95% Confidence interval.

### Multivariate Estimates

When controlling for age at baseline, sex, marital status and instruction level, employment status and alcohol and illicit drugs use, MDEb remained as a significant hazard factor for NO, (HR = 3.54; 95%CI: 1.43–8.75). Of the covariates included in the final model, only Cocaine Basic Paste remained as a significant hazard factor for NO (HR = 3.84; 95%CI: 1.02–14.49). Details are presented in Table 6.

**Table 6:** Model of MDE as hazard factor for Negative outcomes controlling by age, sex, instruction level and marital status.

Variable		HR	P-value	CI 95%
MDE at baseline		3.54	0.006	1.43–8.75
Age at baseline		0.99	0.677	0.95–1.03
Female		0.84	0.746	0.29–2.42
Marital Status	Single	Reference Category		
	Cohabitant/Married	2.22	0.167	0.72–6.85
	Divorced/Widowed	2.5	0.232	0.56–11.3
Instruction level	Incomplete Elementary	Reference Category		
	Complete Elementary	0.97	0.966	0.19–5.02
	Complete High School	1.74	0.503	0.34–8.85
	Superior	1.86	0.497	0.31–11.07
Alcohol Intake	Never	Reference Category		
	Social events only	0.36	0.083	0.11–1.15
	At least onceper month	1.08	0.886	0.36–3.25
Illegal Drugs	Cocaine Basic Paste	3.84	0.047	1.02–14.49
	Cannabis	0.62	0.548	0.13–2.96
Employment	Unemployed	Reference Category		
	Employee	1.08	0.933	0.19–6.03
	Independent Work	0.69	0.507	0.23–2.07

MDE: Major Depressive Episode; HR: Hazard Ratio; CI 95%: 95% Confidence Interval.

## Discussion

Our results showed that MDEb is a significant hazard for negative outcome during TB treatment, and should be addressed before to start TB treatment to improve outcomes. Success on TB control based on DOTS requires 85% cure rate among the detected patients. Peru has a 68% of the patients (new smear-positive) with treatment success, with 5% of defaulting treatment [1], showing the fact of the importance to reduce defaulting proportion as a part of a strategy to achieve success cured rate. Many studies showed multiple risk factor for non-adherence for TB treatment (drug abuse, HIV con-infection, access to treatment, education level, substance abuse and general mental health status) [15], [16], [17], [18] but only a few try to observe the specific role of depression [19], [20]. One retrospective study in the 1990s showed 52.2% of a group of patients with MDR-TB in Lima, Peru had depression [21]. While in our prospective study had a slightly different population (our study evaluated new TB patients with non history of TB, a different population compared with MDR-TB patients, with probably previous TB episodes), we found 37% of patients with MDE at baseline, confirming the high prevalence of depression among TB patients in general.

Although there are several studies showing the importance to study depression in chronic diseases as diabetes and cardiovascular diseases [22], [23], [24], [25], [26], [27], [28] and HIV [29], [30], [31], [32], [33], there is not a specific study looking longitudinal MDE and Tuberculosis treatment outcome. Some studies evaluated mental health among HIV/TB co-infected patients showing the importance of screening mental disorders as part of a comprehensive and holistic clinical approach [34], [35].

The majority of the studies of TB and depression are cross-sectional: One study in South-Africa (60% of HIV co-infection among the study population) showed a 34% of the patients with psychological stress presented non-adherence with TB treatment [20]. Other two cross-sectional studies showed 80% and 72% of prevalence of depression (evaluated with the Beck-scale) and anxiety and depression (evaluated with Hospital Anxiety and Depression Scale - HADS), however these studies had small sample sizes (N = 60 and N = 65 respectively) [19], [36].

A better adherence showed better outcomes and a reduction of drug resistance in the future [37], [38], [39], but a good adherence depend on many factors: An adequate DOTS program and trained personnel in TB treatment is not enough to assure treatment success, because TB have also a social and economical dimension. A systematic review on qualitative studies concluded on 4 main factors for TB adherence: structural factors, social context, health service factors and personal factors [40]. Mental health is affected by these four factors, but the impact of all these factors and how affect mental health status on TB patients should be studied further. Our study showed a first approach about the role of MDE as a factor for treatment success, with an unexpected stronger association than the originally hypothesized.

There are some limitations: one is exclusion of MDR-TB patients (around 10% of the calculated sample size). These MDR-TB patients (considered as primary MDR-TB because is their first episode of TB) were excluded because the diagnosis of MDR-TB can affect the outcome (longer treatment and more severity could lead to a depressive status) and were excluded after enrolment because the drug sensitivity test was available some days after starting treatment. We excluded 12 patients also from the analysis because they decline to participate in the study after enrolment (the main reason was lack of time to fill follow-up CES-D form, no matters take less than 10 minutes to do it), but a sensitivity analysis showed no change in direction of the results (HR: 3.44; 95%CI: 1.4–8.5). Another limitation was a smaller sample size compared with the calculated sample size, and the explanation could be our conservative sample size calculation, considering a HR of 2.0, an assumption of MDE burden among this population: because we don’t have actual data of Mental Health, we extrapolate from a previous study 10 years ago [41], but still showed an strong association. A fourth limitation is unknown confounding, because MDE is also multifactorial, and maybe some of these factors affected the TB treatment outcome (other factors as income, genetics or adverse events for example), however we try to reduce the effect of this limitation with a very homogenous population and the results are consistent with other studies [17], [18].

In conclusion, we found an association between MDE at baseline and negative outcomes (deaths and absconders) to TB treatment in our setting. Our findings indicate that mental health evaluation and treatment of these disorders should be regularly incorporated in the management of TB patients, providing the adequate tools to the clinician to identify and manage these patients at this primary care level.
